# Research stories along the urban water cycle

**DOI:** 10.1016/j.wroa.2024.100218

**Published:** 2024-03-09

**Authors:** Treavor H. Boyer, Wolfgang Gernjak

**Affiliations:** a School of Sustainable Engineering and the Built Environment (SSEBE), Arizona State University, PO Box 873005, Tempe, AZ 85287-3005, USA; b Catalan Institute for Water Research (ICRA), 17003 Girona, Spain; c Catalan Institution for Research and Advanced Studies (ICREA), 08010 Barcelona, Spain

*Water Research X* was launched as a separate journal in January 2023 (Yuan and Morgenroth, 2023) and started publishing articles in its new research narrative format in May 2023 (Boyer and Gernjak, 2023). The scope of *Water Research X* covers the science and technology of the anthropogenic water cycle. This editorial looks at the research articles published in *Water Research X* since May 2023 and the topics that have resonated with authors and reviewers. Specifically, this editorial follows water as it flows through the urban water cycle to highlight research trends published in the journal.

For this story, the urban water cycle begins at wastewater treatment, where technological innovations, environmental impacts, and human activities all converge. For example, advancing the performance of biological wastewater treatment through processes like anammox, granular sludge, and enhanced biological phosphorus removal (An et al., 2023; Nguyen et al., 2023; Wen et al., 2023; Yin et al., 2023; Zhang et al., 2024; Zheng et al., 2023) reflect the need for wastewater plants to do more. Wastewater treatment advancements also benefit from understanding the interactions of microbial communities (Gao et al., 2023; Kim et al., 2023), and research on underexplored areas such as microbial predation and parasitism (Kuroda et al., 2023). In addition to centralized wastewater treatment, decentralized treatment also needs innovative solutions, such as stormwater and rainwater harvesting and nutrient recovery from waste streams (Keller, 2023). And as wastewater treatment plants do more, it is important to quantify the impacts of wastewater operations on the environment such as greenhouse gas emissions (Bai et al., 2023) and sewer integrity (Zhou et al., 2023). Finally, the constituents in wastewater provide a window to the communities that generate the wastewater such as use of psychoactive substances (Bade et al., 2023).

Following wastewater discharge, and sewer overflows, into the natural aquatic environment (Furrer et al., 2023), microplastics have captured considerable attention. Research challenges include detecting microplastics and nanoplastics (Chen et al., 2023), and quantifying their interactions with natural minerals and contaminants (He et al., 2023). Stormwater runoff also enters the natural aquatic environment, with research investigating strategies to mitigate release for per- and polyfluoroalkyl substances (Vo et al., 2023) and phosphorus interactions with sediment (Wang et al., 2023). Research on the natural aquatic environment goes beyond freshwater quality to include coastal ecosystems (Lo et al., 2023) and the availability of water resources influenced by climate change and drought (Penny et al., 2023).

Surface water, groundwater, and seawater serve as sources of water for communities. Drinking water treatment technologies need improved ways to treat diverse water sources and remove legacy and emerging contaminants. For example, capacitive deionization for brackish water desalination (Tan et al., 2023) and designing new adsorbents for per- and polyfluoroalkyl substances (Yan and Liu, 2023). There is also the need for new and improved ways to inactivate microorganisms such as UV LEDs (Uppinakudru et al., 2023) and photocatalytic membranes (Zhong et al., 2023). Hot water is an important use of potable water in homes and buildings where it consumes considerable energy with associated greenhouse gas emissions (Kenway et al., 2023) and can harbor opportunistic pathogens in hot water pipes of different materials (Cullom et al., 2023), and the pathogen risk of building water systems can be worsened by stagnant water conditions (Huang et al., 2023). Finally, many physical-chemical water treatment technologies have been adapted for wastewater treatment, especially waste streams like human urine and the details of pharmaceutical adsorption to activated carbon (Heusser et al., 2023).

An increasingly important component of the urban water cycle is water reuse, whether for agriculture (Yalin et al., 2023), industrial (Choi et al., 2024), or human consumption (Mackey et al., 2023). We welcome authors to submit their research from across the urban water cycle and look forward to the important stories that the research tells.

## CRediT authorship contribution statement

**Treavor H. Boyer:** Writing – original draft, Conceptualization. **Wolfgang Gernjak:** Writing – review & editing, Conceptualization.

## Declaration of competing interest

The authors declare that they have no known competing financial interests or personal relationships that could have appeared to influence the work reported in this paper.

## Data Availability

No data was used for the research described in the article
